# World Heart Day 2025 in Prasanthi Nilayam, India: A Silver Jubilee of the World Heart Federation Advocacy at the Centenary of a Humanitarian Legacy

**DOI:** 10.5334/gh.1567

**Published:** 2026-07-28

**Authors:** Nikhila Sri Rao, Borjana Pervan, Viswanathan Mohan, Krishna M. Rao, Anil Kumar Mulpur, Antonio Bayés de Luna, Sidney C. Smith, Jagat Narula, On behalf of the World Heart Federation, Geneva, Switzerland and Sri Sathya Sai Central Trust, Puttaparthi, India

**Affiliations:** 1 State University of New York Upstate Medical University, New York, United States; 2 World Heart Federation, Geneva, Switzerland; 3 Dr. Mohan’s Diabetes Specialities Centre & Madras Diabetes Research Foundation, Chennai, India; 4 University of Rochester Medical Center, Rochester, NY, United States; 5 Sri Sathya Sai Global Council USA, United States; 6 Sri Sathya Sai Institute of Higher Medical Sciences, Prasanthigram, Andhra Pradesh, India; 7 Cardiothoracic and Vascular Surgery, Sri Sathya Sai Institute of Higher Medical Sciences, Prasanthigram, Andhra Pradesh, India; 8 Past President of the World Heart Federation, Spain; 9 University of North Carolina, Chapel Hill, NC, United States; 10 University of Texas Health Houston, Houston, Texas, United States; 11 McGovern Medical School, University of Texas Health Houston, Houston, Texas, United States

**Keywords:** World Heart Day 2025, cardiovascular disease prevention, global heart health

## Abstract

**An Unprecedented Confluence of Science, Selfless Service, and the World Heart Federation’s Mission of Cardiovascular Health for All**

On 29 September 2025, the World Heart Federation (WHF) marked the **silver jubilee**—25 years—of World Heart Day in what might seem an unlikely setting for a global cardiovascular organization: Prasanthi Nilayam in Puttaparthi, India, an internationally recognized epicenter of free, world-class medical care and humanistic service. The choice was deliberate. For an organization whose mission is ‘cardiovascular health for everyone, everywhere,’ it was most fitting to celebrate this milestone in a place where that principle has been quietly practiced, at scale and at no cost, for decades.

For most of its history, the WHF has marked World Heart Day through campaigns centered initially only in its headquarters in Geneva, and subsequently in capitals and at multilateral fora, where global policy is debated and shaped. By contrast, Prasanthi Nilayam sits closer to village life than to ministries of health, and closer to patients’ daily realities than to conference halls. Precisely for that reason, it offered a real-life model of how values-driven policy translates into free, high-quality cardiovascular care, prevention, and community engagement on the ground.

The timing was equally symbolic: the 25th anniversary of World Heart Day coincided with the centenary celebrations of Sri Sathya Sai Baba, whose guiding maxim—‘Love All, Serve All; Help Ever, Hurt Never’—has, for decades, animated an integrated, free-of-cost healthcare ecosystem that mirrors the WHF’s founding pledge that cardiovascular health should be a right, not a privilege, regardless of background, religion, caste, or means.

## From the International Society of Cardiology to the World Heart Federation: A Brief History

The WHF traces its roots to the immediate post-war years, when cardiology first organized itself as a global, civic enterprise. Its evolution from a small federation of national societies to a 220-member global advocate mirrors the rise of cardiovascular disease (CVD) as the world’s leading killer and the simultaneous determination of the cardiology community to confront it together.

Founded in **1946** with seven national cardiology societies, the *International Society of Cardiology (ISC)* was conceived as a scientific congress of professionals. A quarter century later, in **1970**, a parallel body—the *International Cardiology Federation (ICF)*—was established by heart foundations from around the world to advance research, public education, and community programs. In **1978**, these two organizations merged to form the *International Society and Federation of Cardiology (ISFC)*, joining the scientific and civic missions under one banner. Two decades later, at the World Congress of Cardiology in Rio de Janeiro in **1998**, the Board renamed the organization the **‘World Heart Federation,’** reflecting its expanded global reach and its commitment to public-health advocacy beyond the academic clinic. The WHF today partners with the WHO, UNESCO, and governments worldwide, works through more than 220 member organizations, and serves as the international voice of cardiovascular health.

**Figure:**
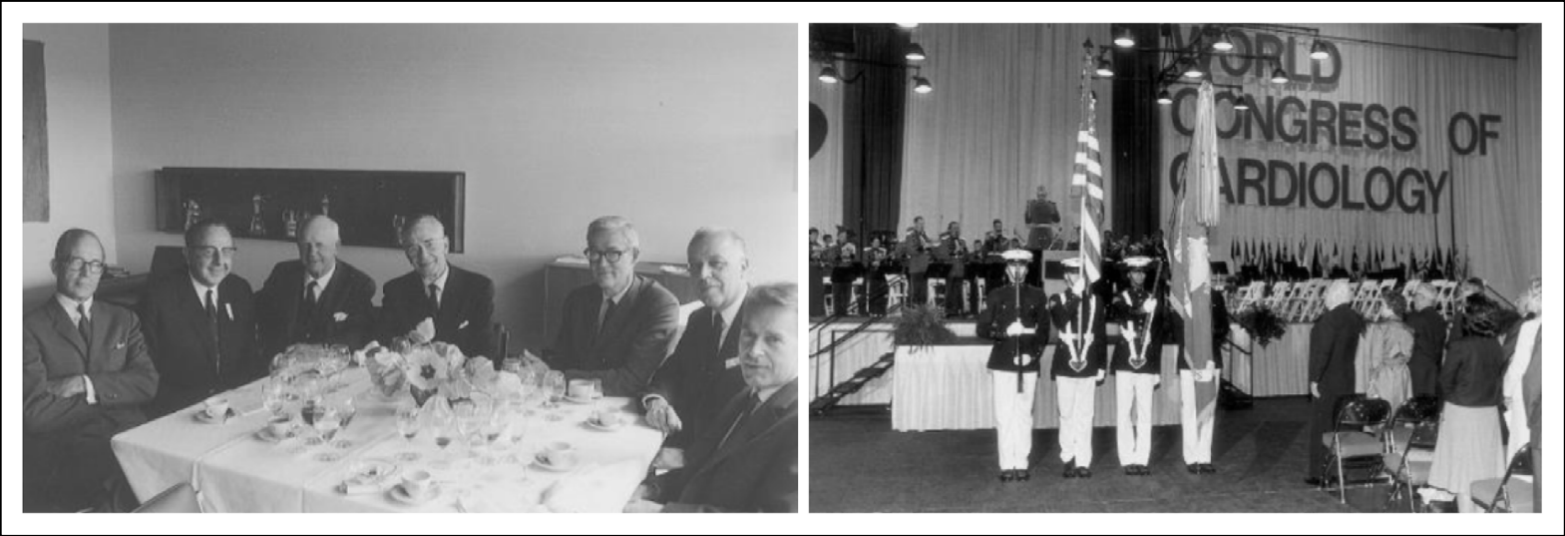
**History of the World Heart Federation.** The WHF traces its origins to the 1978 merger of the ISC and the ICF, later adopting the name ‘World Heart Federation’ during the World Congress of Cardiology in Rio de Janeiro, 1998. Today, the WHF collaborates with more than 220 members from across the globe.

**Table:** 


YEAR	MILESTONE

**1946**	The International Society of Cardiology (ISC) is founded as a professional scientific organization with seven national cardiology societies.

**1970**	The International Cardiology Federation (ICF) is established, uniting heart foundations to support international research, professional and public education, and community programs.

**1978**	The ISC and ICF merge to form the International Society and Federation of Cardiology (ISFC), creating a unified global voice for cardiology.

**1990s**	The ISFC works in partnership with the WHO and UNESCO on heart disease and rheumatic fever prevention initiatives.

**1998**	At the World Congress of Cardiology in Rio de Janeiro, the ISFC Board renames the organization the ‘World Heart Federation.’

**1999**	Under President Antonio Bayés de Luna, MD, the WHF conceives a global awareness day to bring CVD into the public conversation.

**2000**	The first World Heart Day is celebrated on 24 September in partnership with the WHO under the slogan ‘Let It Beat,’ highlighting the benefits of physical activity.

**2001**	The WHF launches its first formal mission statement, focused on the prevention and control of heart disease and stroke in low- and middle-income countries.

**2011**	World Heart Day is permanently fixed to be on 29 September each year.

**2022**	The WHF launches the World Heart Observatory, a global portal of CVD data and knowledge.

**Today**	The WHF works with more than 220 member organizations across the globe—the cornerstone of cardiovascular health advocacy.


## World Heart Day: 25 Years of a Global Movement

World Heart Day was the vision of **Antonio Bayés de Luna, MD**, President of the WHF from 1997 to 1999. Concerned that CVD—already the leading cause of death worldwide—lacked a unifying day of public attention, he proposed an annual global event to translate cardiology’s science into civic awareness. The first **World Heart Day** was held on **24 September 2000** in partnership with the WHO, under the inaugural slogan ‘*Let It Beat*,’ celebrating the benefits of physical activity. For its first decade, the day was observed on the last Sunday of September; since **2011**, it has been permanently anchored on **29 September**, uniting more than 100 countries on a single date of advocacy.

**Figure:**
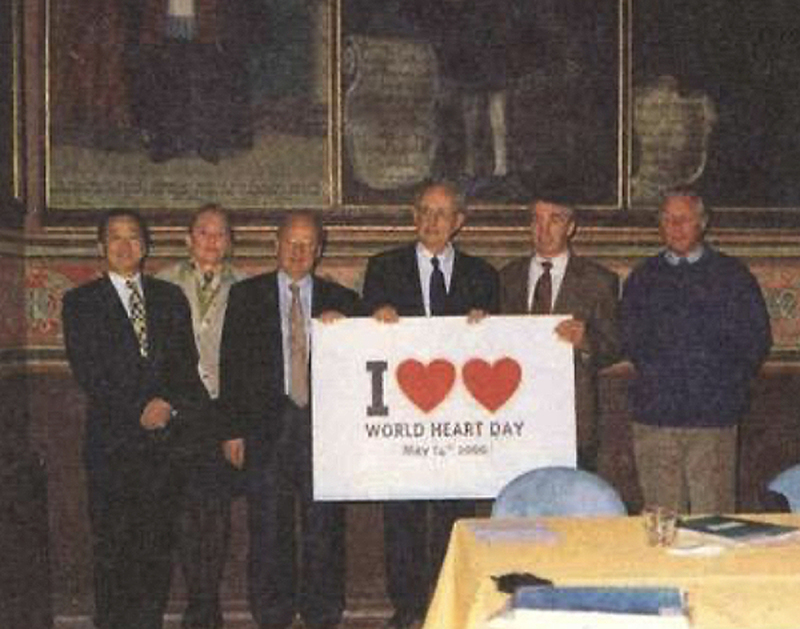
**Celebrating the first World Heart Day Campaign.** Launched on 24 September 2000 under the slogan ‘Let it Beat,’ the first World Heart Day was celebrated in partnership with the WHO to encourage international action and awareness of CVD. Pictured left to right: Tak-Fu-Tse, Marianne Burle de Figueiredo, John Chalmers, Antonio Bayés de Luna (WHF President, 1997–1999), Darwin R. Labarthe, and Leslie Busk.

Each year, a new theme is chosen—from **‘Women, Heart Disease and Stroke’** in 2003, to **‘Know Your Risk’** in 2008, to the multi-year **‘Use Your Heart’** or the simply-put **‘Use** ♥**’** campaign launched in 2020—framing the conversation around the most pressing cardiovascular health questions of the day.

The 2025 theme, ***‘Don’t Miss a Beat,’*** calls on every individual to recognize the early warning signs of heart disease, maintain continuous access to care, and treat heart health as a daily priority. Beyond individual awareness, the 2025 campaign also carried a clear policy ask. In the same month, global leaders convened at the UN High-Level Meeting on Non-Communicable Diseases and Mental Health, reinforcing the urgent need for action on non-communicable diseases, with CVD at the forefront of discussions. The World Heart Day message and WHF advocacy work were deliberately aligned to that moment, calling on governments to strengthen prevention, primary care, and equitable access to cardiovascular services.

**Figure:**
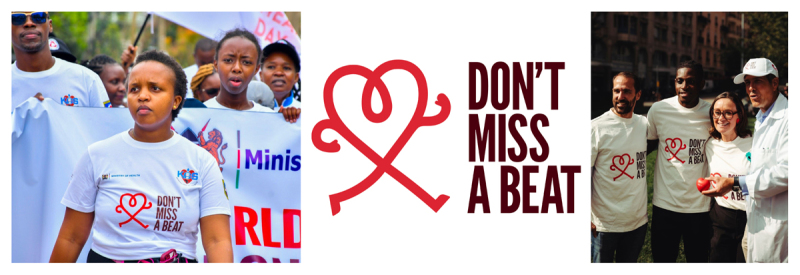
**World Heart Day 2025 Campaign ‘Don’t Miss a Beat.’** Uniting voices worldwide to champion prevention, timely action, and equitable heart care for all.

World Heart Day now reaches an estimated 1.7 billion people each year through the WHF’s member-driven efforts—from zumbathons, walks, runs, and hypertension screening camps to roundtables, drawing contests, media activities, social-media campaigns and the illumination of landmarks in red—from Niagara Falls and the Eiffel Tower to the Empire State Building. Across an unprecedented engagement of more than 130 member organizations, this diversity of local action turns a single global theme into thousands of community-level moments of engagement.

**Figure:**
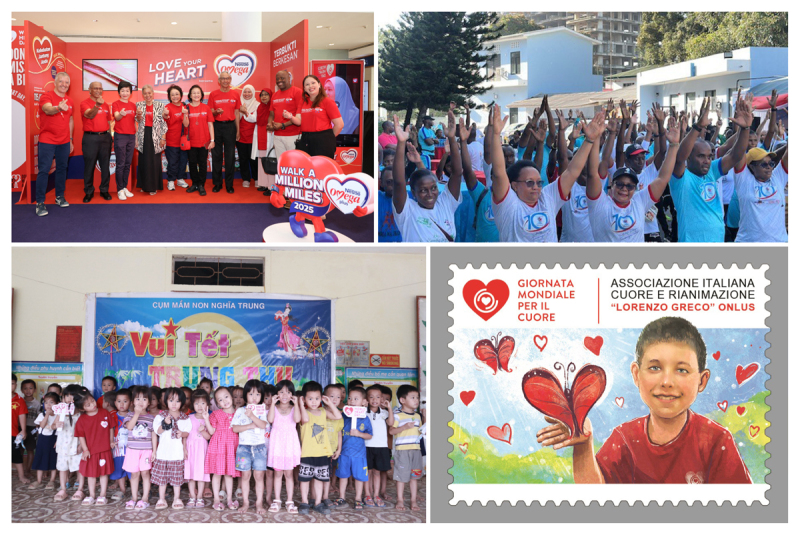
**World Heart Day.** The day has symbolized a global commitment to cardiovascular education and collective action, empowering people from all walks of life to value, protect, and prioritize heart health.

**Table:** Twenty-Six Themes, One Heartbeat.


YEAR	WORLD HEART DAY THEME

**2000**	Let It Beat — Physical Activity

**2001**	A Heart for Life

**2002**	What Shape Are You In?

**2003**	Women, Heart Disease and Stroke

**2004**	Children, Adolescents and Heart Disease

**2005**	Healthy Weight, Healthy Shape

**2006**	How Young Is Your Heart?

**2007**	Team Up for Healthy Hearts

**2008**	Know Your Risk!

**2009**	Work with Heart

**2010**	Workplace Wellness — I Work with Heart

**2011**	One World, One Home, One Heart

**2012**	One World, One Home, One Heart

**2013**	Take the Road to a Healthy Heart

**2014**	Heart Choices, Not Hard Choices

**2015**	Creating Heart-Healthy Environments

**2016**	Power Your Life

**2017**	Share the Power

**2018**	My Heart, Your Heart

**2019**	My Heart, Your Heart

**2020**	Use ♥ to Beat Cardiovascular Disease

**2021**	Use ♥ to Connect

**2022**	Use ♥ for Every ♥

**2023**	Use ♥, Know ♥

**2024**	Use ♥ for Action

**2025**	Don’t Miss a Beat — Silver Jubilee


## World Heart Day 2025: The Setting at Prasanthi Nilayam

Rather than a conventional congress center or capital city, World Heart Day 2025 unfolded in Prasanthi Nilayam in Puttaparthi, India—a township built around world-class, free medical care and a long-standing culture of selfless service. The celebrations represented an unprecedented collaboration between the WHF and the Sri Sathya Sai Institute of Higher Medical Sciences (SSSIHMS) Prasanthigram and Whitefield Hospitals, College of Nursing at SSSIHMS, Sri Sathya Sai General Hospital (SSSGH), Sri Sathya Sai Mobile Hospital, Sri Sathya Sai Institute of Higher Learning (SSSIHL, all campuses), Sri Sathya Sai Primary and Higher Secondary Schools, Smt. Eswaramma High School, Sri Sathya Sai Seva Organization (SSSSO), Sri Sathya Sai Media Centre (SSSMC), Sai Hira Global Convention Centre, Sri Sathya Sai Global Council (SSSGC), and sister institutions under the Sri Sathya Sai Central Trust (SSSCT).

Set in a rural region that once lacked any formal health services, Prasanthi Nilayam has evolved into a living demonstration of how high-quality cardiovascular care, prevention, and follow-up can be delivered free of charge, close to where people live. For the WHF, holding the Silver Jubilee here meant putting its advocacy principles into practice: observing and supporting a model in which access, equity, and dignity are not abstract policy goals but the organizing principles of daily care.

The timing added a further layer of meaning. World Heart Day’s 25th anniversary coincided with the centenary celebrations of Sri Sathya Sai Baba, a spiritual teacher whose maxim—‘Love All, Serve All; Help Ever, Hurt Never’—continues to shape the institutions of Prasanthi Nilayam. Over several decades, that ethos has underpinned an integrated, free-of-cost healthcare system that treats cardiovascular health as a right rather than a privilege and actively reaches people across the lines of background, religion, caste, and means.

Love All, Serve All; Help Ever, Hurt Never.

— The guiding principle of Prasanthi Nilayam, and a fitting motto for cardiovascular health for all

Spanning three days from **27 to 29 September 2025**, World Heart Day at Prasanthi Nilayam unfolded as a multifaceted initiative that brought clinical care, academic dialogue, and community outreach to a *grassroots* level. The program comprised a community-based free medical camp, a global preventive cardiovascular conference, a student-led preventive-cardiology scientific exhibition, a public heart walk culminating in a striking human heart formation, and an evening cultural drama—all aligned to the 2025 WHF theme of ***‘Don’t Miss a Beat’*** and the overarching local theme of ***‘Preventive Cardiology: Reducing Hurry, Worry, and Curry.’*** Iconic landmarks were illuminated in red, symbolizing a collective resolve toward cardiovascular health. Attended by medical professionals, trainees, public-health advocates, students, dignitaries, scientists, and leaders in medicine from across the world, this gathering reflected solidarity and a shared global commitment to translating cardiovascular science, advocacy, and awareness into meaningful, community-level action.

## Day 1 — Community-Based Medical Camp: Translating Awareness into Action

World Heart Day 2025 commenced with a community-based medical camp that provided compassionate medical care, at no cost, to patients from nearby rural communities. On World Heart Day itself, more than 350 patients were screened for diabetes, hypertension, and cardiovascular risk factors. Through continued outreach thereafter, as many as 28,000 patients were tended to and screened for common CVD risk factors.

Cutting-edge services were offered, including point-of-care ultrasound (POCUS), AI-empowered EKG, glucose tolerance testing, free referrals to CT coronary calcium scoring at the nearby charity hospital, and comprehensive on-site patient counseling regarding nutrition, hands-only CPR, smoking cessation, stress management, and physical activity. Through the holistic nature of these services, cardiovascular advocacy and prevention were not merely matters of speech or rhetoric—they were awareness translated into action.

**Figure:**
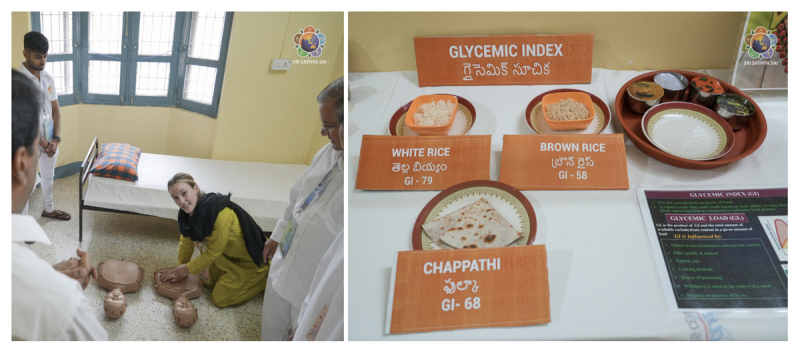
**Preventing Cardiovascular Disease through Community Empowerment.** From hands-only CPR demonstrations to nutritional counseling, this community medical camp emphasized that heart health begins with awareness, action, and accessible care.

The entire medical camp was driven by selfless effort from nurses, doctors, nutritionists, diabetes specialists, allied health professionals, sonographers, medical students, and kind-hearted volunteers. Patients of all age groups were evaluated for indications such as chest pain (CAD-related and non-cardiac), arrhythmias, valvular heart disease (particularly rheumatic heart disease), and congenital heart disease, including ventricular septal defect, Eisenmenger’s syndrome, bicuspid aortic valve disease, and prosthetic valves. The camp was organized into the following departments: Registration, Triage (vitals), Lab Work, Cardiology (equipped with EKG and POCUS), Diabetes, Nutrition & Patient Counseling, Emergency (equipped with AEDs), and Pharmacy.

### ‘All-in-One Cardiac Care’ — EKG and POCUS at the Doorstep

The introduction of EKG and POCUS modalities into a community medical camp could be coined ‘All-in-One Cardiac Care.’ POCUS led to expeditious diagnoses and management of patients, many of whom lacked continuous access to healthcare once they returned to their home villages. The portable ultrasound device helped evaluate numerous patients presenting with acute concerns of chest pain, dyspnoea, palpitations, abdominal pain, and head and neck swelling.

To illustrate: a middle-aged man presented with congestive heart failure, likely triggered by new-onset atrial fibrillation discovered at the camp. Handheld POCUS, combined with a comprehensive examination, led to a prompt diagnosis and—most importantly—the prevention of possible stroke and worsening of acute heart failure. The patient was started immediately on life-saving medications, including anticoagulation, and was referred to an inpatient setting for further care. He hailed from a distant rural village and stated that he had never seen a doctor before, owing to financial hardship and geographical barriers. Without the rapid, timely diagnostics and community-centered care offered through this camp, he would have suffered grave complications.

**Figure:**
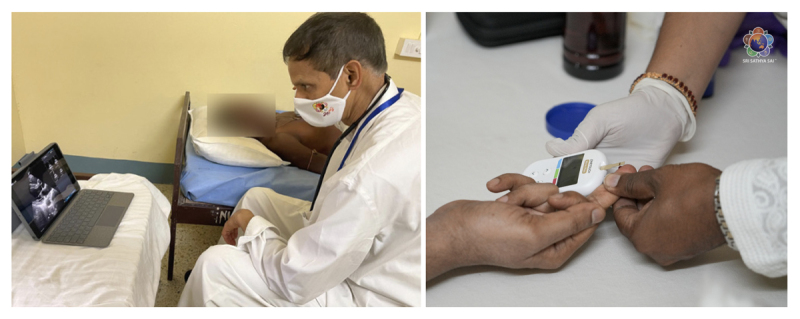
**Advancing Early Detection in Community Heart Health.** This medical camp integrated diagnostic EKGs, POCUS imaging, and cardiometabolic screening to support early identification of cardiac pathology and risk stratification.

Alongside the impactful care delivered to patients, the medical camp fostered a spirit of unity and an environment conducive to collaboration and teaching. For every patient treated, a coordinated team of volunteers, technicians, students, and healthcare personnel was actively involved—from registration through triage, phlebotomy, diagnostics (EKG/POCUS), and the pharmacy pickup. Patient flow was meticulously organized, leading to smooth and efficient assessments. The exercise demonstrated the immense power of bringing care to the doorsteps of individuals within their own communities and serving the hearts that often go unseen—truly, ‘don’t miss a beat.’

## Days 2 & 3 — Sai Global Cardiovascular Symposium: Knowledge without Borders

The second day of the World Heart Day celebrations marked the inauguration of the **Sai Global Cardiovascular Symposium**—a two-day academic conference held at the state-of-the-art *Sai Hira Global Convention Center* on-site in Prasanthi Nilayam. Approximately 65 leading clinicians in cardiovascular medicine, esteemed researchers, and public-health leaders convened to examine emerging directions and contemporary challenges in cardiovascular care, prevention, and advocacy.

The two-day conference comprised morning and afternoon scientific sessions addressing a wide variety of topics in preventive cardiology, advances in diagnostic and therapeutic strategies, and the role of community-based approaches. Speakers gave their perspectives on the magnitude of the global cardiovascular burden, traditional risk factors such as diabetes, hypertension, and stress, and emerging risk factors such as air pollution, sedentary lifestyle, and inflammation. The program concluded with talks on holistic approaches—stress management, yoga, meditation, and dietary strategies to mitigate disease. The symposium was framed around the shared global burden of CVD while acknowledging the heterogeneity in risk profiles, healthcare infrastructure, and outcomes across countries.

**Figure:**
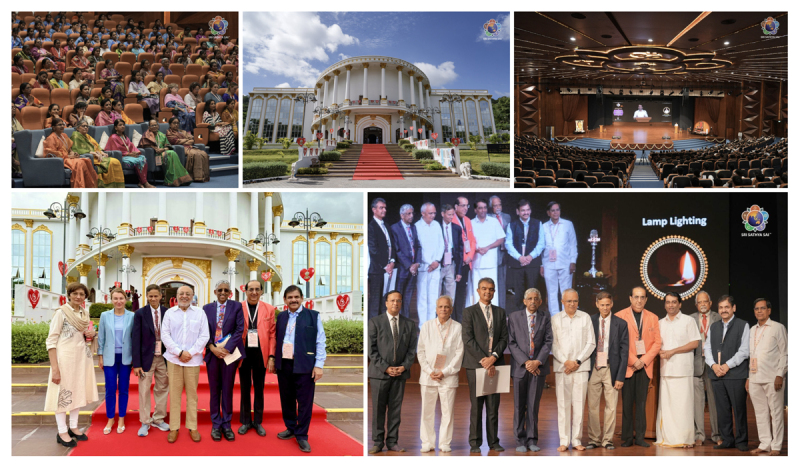
**Sai Global Cardiovascular Symposium.** The two-day conference brought together thousands of delegates from around the world, alongside prominent speakers and leaders in cardiovascular science.

**Selected Presentations** included Diabetes in Indians; Hypertension Update 2025; Stress and Heart Disease- Broken Heart Syndrome, SCAD, and Coronary Spasm; Global Remote Medical Camps; Rheumatic Valvular Heart Disease; Early Detection and Managing Plaque That Matters; Primordial Prevention of CVD; Precision Medicine to Improve Safety and Outcomes; Secondary Prevention Following Myocardial Infarction; Well-being, Stress, Burnout, Depression, and Cardiovascular Health; Heart-Healthy Dietary Strategies in Rural Settings; and the Lifestyle Prescription for a Healthier Heart. The chief speaker and guest of honor, **Dr. Jagat Narula, MD, PhD**—president of the WHF—presented the global cardiovascular disease burden in his keynote, calling for both on-the-ground prevention campaigns and population-level healthcare reform. He underscored the significance of WHF’s 2025 sub-theme, **‘Don’t Miss a Beat,’** urging individuals and policymakers alike to make heart health a universal priority so that ‘every beat counts for every heart.’

The conference also included insightful panel sessions on electrophysiology, interventional cardiology, cardiothoracic surgery, echocardiography, and service-based medical outreach, as well as a practical demonstration of hands-only CPR by a team of cardiac nurses. Presenters and audience members alike were diverse—cardiologists, diabetologists, general medicine practitioners, surgeons, sonographers, registered dietitians, nurses, engineers, medical students, and doctoral researchers—reflecting the breadth of disciplines now needed to advance cardiovascular health. The Sai Global Cardiovascular Symposium served as a platform for fruitful dialogue, exchange of knowledge, the introduction of novel ideas, and an updated picture of where cardiovascular prevention stands today.

## Voices of the Next Generation

Parallel to the community medical outreach and academic didactics, a defining feature of World Heart Day 2025 in Prasanthi Nilayam was the integration of cultural and artistic modalities led by students. It was through the lens of the youth and the rising next generation that the message of cardiovascular health reached the broader public in a universal, inclusive, and meaningful way. These initiatives demonstrated a simple truth: for prevention to be sustainable, it requires engagement that speaks to people’s emotions, narratives, and shared identities.

**Figure:**
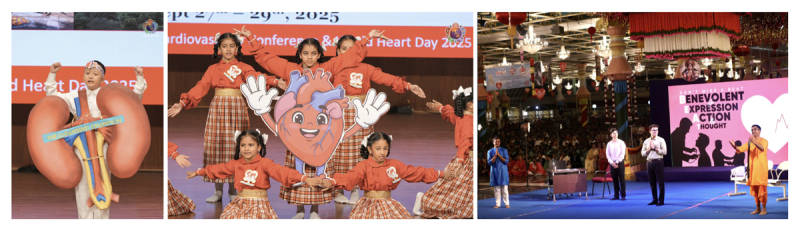
**Hearts in Harmony.** Primary school and university students present impactful cultural dramas celebrating heart health, wellness, and compassion. Don’t miss a ‘BEAT’—being **b**enevolent in **e**xpression, **a**ction, and **t**hought!

## Student-Led Heart Health Exhibition

Approximately 1,500 students—from elementary school through university and post-graduate level—came together to mount a walk-through health exhibition designed to educate the public on CVD and prevention. The topics included nutrition, physical activity, tobacco cessation, stress management, causes and types of heart disease, and glycemic control in diabetes, among many others.

Students created hundreds of handmade and digital posters, board games, three-dimensional models, puzzles, online quizzes, short live drama and dance presentations, original songs, short films, iPhone apps, and interactive electronic models. Their presentations were curated from a thorough review of the literature and evidence-based cardiovascular guidelines and rendered in a culturally intelligible and visually accessible format. Analogies, themes, and narrative storytelling were used to convey CVD prevention to an audience of diverse backgrounds and varied health literacy. The students’ work was not only academically sound—it was also threaded through with positive affirmations and encouraging messages that motivated the public.

**Figure:**
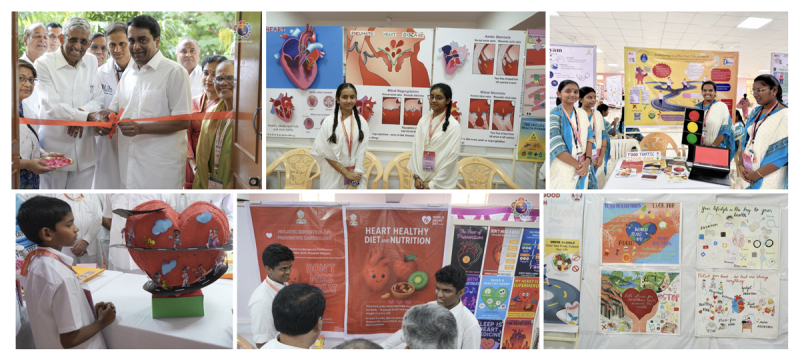
**The Future of Heart Health.** Students present an inspiring showcase of projects and innovative ideas dedicated to CVD prevention and education.

This exhibition was a highly successful and impactful educational event for both students and visitors from all walks of life. The discipline, orderliness, enthusiasm, and depth of knowledge among the participating students were palpable. Many of them came from families of auto-rickshaw drivers, farmers, and parents who had little or no opportunity for schooling. Their families often face challenging life circumstances such as alcoholism and financial hardship. The learning experience and exposure created excitement among the students to share their newfound knowledge with their families at home. The event served as a reminder of how instrumental children and youth are in disseminating advocacy, promoting health literacy, and inspiring real change. Empowering students means empowering families, communities, and ultimately countries.

## Heart Walk and the Human Heart Formation

Early on World Heart Day (29 September), students, conference delegates, WHF representatives, and the public assembled for a community heart walk. Everyone was uniformly dressed in bright red ‘World Heart Day 2025’ T-shirts, waving colorful banners and holding vibrant placards with heart-healthy messages. The walk concluded at the Sri Sathya Sai Hill View Stadium, where participants were greeted by a spectacular human heart formation created by students. A second group of students formed the WHF logo—a celebration of the global commitment to cardiovascular awareness, prevention, and advocacy. The Heart Walk and the human formations conveyed the instrumental role of community in promoting heart-healthy behaviors such as physical activity and stress management.

**Figure:**
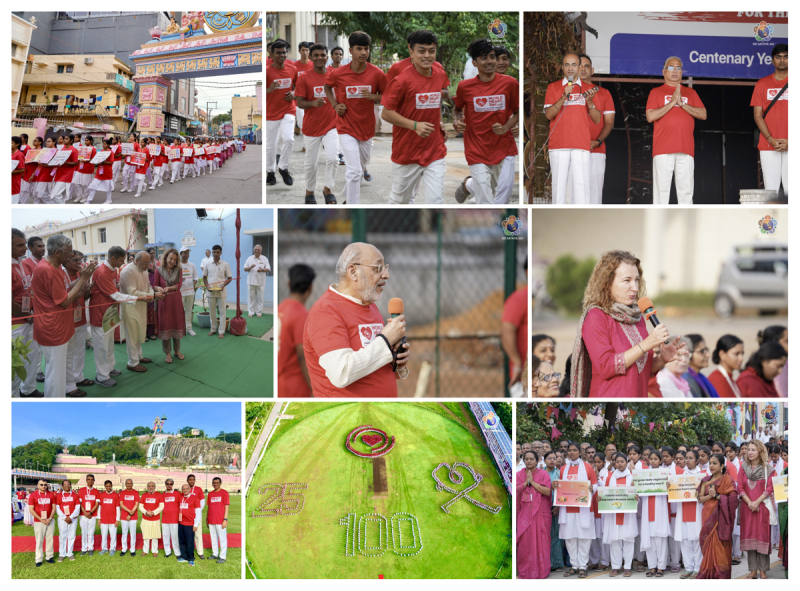
**World Heart Day 2025 Celebrations in Prasanthi Nilayam, India.** A community heart walk for heart health awareness, culminating in a striking heart formation by the students.

## Shine for World Heart Day

As part of World Heart Day 2025, the SSSIHMS and the SSSGH—hospitals long recognized for delivering high-quality, accessible care completely free of cost—were illuminated in red, joining the WHF tradition of lighting iconic landmarks across the globe (Niagara Falls, Eiffel Tower, Empire State Building) on this day each year. The red illumination at Prasanthi Nilayam was not only a symbolic gesture but also a public message: that everyone, irrespective of background, religion, socio-economic status, or caste, is deserving of compassionate cardiovascular care.

**Figure:**
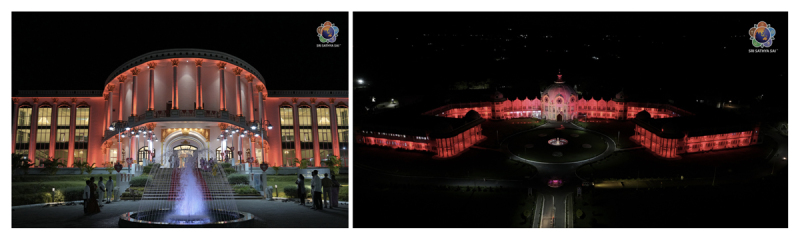
**Red Illumination for World Heart Day.** The Sai Hira Global Convention Center and Hospitals (SSSIHMS, SSSGH) illuminate in red, marking solidarity with the WHF’s global movement and call for cardiovascular disease prevention and health promotion.

## Conclusion — A Mission Renewed

World Heart Day 2025 left an unforgettable impact on all who participated in person and the millions who tuned in from around the world. World Heart Day 2025 in Prasanthi Nilayam, India, illustrated the power of cardiovascular advocacy through a combined approach of academic discourse, community medical outreach, and memorable public engagement led, in large part, by the rising generations.

In its silver-jubilee year, World Heart Day returned to a first principle: that cardiovascular health is not the privilege of a few but the right of all—the very founding promise of the WHF. By holding the day at a place where world-class medical care has been provided free of cost for decades, and by anchoring it to the centenary of a humanitarian whose maxim was ‘Love All, Serve All,’ WHF was able to show, in one setting, how its global advocacy and policy asks—from the UN to national ministries—can translate into concrete models of care on the ground. In doing so, it reaffirmed its mission with both clarity and conscience: **cardiovascular health for everyone, everywhere**

Don’t Miss a Beat — because every beat counts for every heart.

— World Heart Day 2025

## Credit Info

Photographic materials courtesy of Sri Sathya Sai Media Centre, Prasanthi Nilayam, India, under the Sri Sathya Sai Central Trust.

